# Prevalence and morphology of lingual canals in mandibular incisors: a cone-beam computed tomography-based cross-sectional study

**DOI:** 10.7717/peerj.21617

**Published:** 2026-08-05

**Authors:** Cicero Lucas Gomes Ramalho, Eliane Maria Gonçalves Moreira de Vasconcelos, Victor Archeti Vardiero, Cesar Rogério Pucci, Amjad Abu Hasna, Milton Santamaria Jr

**Affiliations:** 1 Dentistry, University Center of the Hermínio Ometto Foundation–FHO, Araras, São Paulo, Brazil; 2 Radiological Imaging Center, CECAPE College, Juazeiro do Norte, Ceará, Brazil; 3 Department of Restorative Dentistry, Institute of Science and Technology, São Paulo State University (ICT-UNESP), São José dos Campos, São Paulo, Brazil; 4 Department of Restorative Dentistry, Endodontics Division, Institute of Science and Technology, São Paulo State University (ICT-UNESP), São José Dos Campos, São Paulo, Brazil; 5 Department of Social and Pediatric Dentistry, Institute of Science and Technology, São Paulo State University (ICT-Unesp), São José dos Campos, São Paulo, Brazil

**Keywords:** Endodontics, Incisor teeth, Pulp cavity, Anatomy, Cone beam computed tomography

## Abstract

**Background:**

Accurate identification of root canal anatomy is essential for endodontic success, particularly in mandibular incisors, where additional canals such as the lingual canal are frequently missed using conventional diagnostic methods. In this context, cone-beam computed tomography (CBCT) has emerged as a valuable tool for detecting complex anatomical variations. Therefore, this study evaluated the prevalence, distribution, and spatial characteristics of the lingual canal using CBCT, as well as its association with demographic factors.

**Methods:**

This observational, cross-sectional, retrospective study included CBCT scans from 342 patients (1,368 mandibular incisors). Images were acquired using standardized parameters (field of view: 10 × 5 cm; voxel size/slice thickness: 0.5 mm; interval: 1/1) and analyzed by a single previously trained examiner. Intra-rater reliability was assessed using test–retest analysis in 30% of the sample, demonstrating excellent agreement (ICC = 0.95). Associations between the presence of the lingual canal and demographic variables were evaluated using chi-square tests and odds ratios, while spatial characteristics were assessed using multivariate analysis of variance (MANOVA), adopting a significance level of *p* < 0.05.

**Results:**

The overall prevalence of the lingual canal was 8.84%. It was significantly more frequent in males than females (*p* < 0.05) and was associated with age, with higher occurrence in individuals aged 40–59 years (*p* < 0.01). A higher prevalence was observed on the right side, and the predominant canal configuration was straight. Multivariate analysis showed no significant effect of sex, canal position, or their interaction on the combined spatial variables (distance and angle) (*p* > 0.05). Therefore, the lingual canal is a relatively frequent anatomical variation, more prevalent in males, middle-aged individuals, and on the right side. These findings highlight the importance of careful anatomical assessment to improve endodontic outcomes.

## Introduction

The anatomy of the root canal system plays a critical role in the success of root canal treatment. A thorough understanding of its complexity is essential (Ferrari, Abu Hasna & Martinho, 2021; Henrique Ferrari & Abu Hasna, 2022; Castillo Marin et al., 2024), as failure to recognize anatomical variations may lead to complications such as inadequate instrumentation and obturation, perforations, ledge formation, and instrument fracture (Abu Hasna et al., 2020; Al-Nahlawi, Rachi & Abu Hasna, 2021; Dos Reis et al., 2023; Ragozzini et al., 2024), ultimately resulting in treatment failure (Krasner & Rankow, 2004; Mashyakhy, 2019).

Mandibular incisors are often mistakenly considered simple to treat, as they are commonly assumed to have straight roots and a single canal. However, studies have shown that up to 45% of these teeth present significant anatomical variations, including the presence of a second canal, also described as an additional canal in the literature and referred to throughout this study as the lingual canal (Paes da Silva Ramos Fernandes et al., 2014; Saati et al., 2018; Lee & Seo, 2022). These variations represent substantial challenges for endodontic treatment and often require advanced technical skills (Mashyakhy, 2019; Baxter, Jablonski & Hülsmann, 2020).

The presence of a lingual canal, typically located below the cingulum, increases the complexity of access cavity preparation, instrumentation, and obturation (Ahmed et al., 2024). During treatment, the buccal canal is often adequately instrumented, while the lingual canal may be overlooked, leaving residual pulp tissue. This can negatively affect clinical prognosis (Rahimi et al., 2013; Assadian et al., 2016). Comparative studies have shown that apical periodontitis may be up to 6.25 times more prevalent in teeth with untreated root canals (Costa et al., 2019).

Accurate identification of root canal anatomy remains a critical challenge in endodontics, particularly in mandibular incisors, where anatomical variations such as the presence of a lingual canal may go undetected (Mashyakhy, 2019; Martins et al., 2017). Conventional diagnostic methods, including periapical radiography and clinical exploration under magnification, are widely used but present inherent limitations, especially due to their two-dimensional nature and the superimposition of anatomical structures (Ferrari, Abu Hasna & Martinho, 2021).

In this context, cone-beam computed tomography (CBCT) has emerged as a valuable imaging modality, providing three-dimensional visualization that enhances the detection of additional canals and complex anatomical configurations (Candeiro et al., 2019; Ferrari, Abu Hasna & Martinho, 2021). However, CBCT also presents limitations, including radiation exposure, cost, and resolution constraints that depend on voxel size (Henrique Ferrari & Abu Hasna, 2022). Clinically, its use is particularly indicated in cases of suspected anatomical complexity, treatment failure, or when conventional methods are inconclusive (Candeiro et al., 2019; Martins, Worldwide Anatomy Research Group & Versiani, 2023). Therefore, understanding both the capabilities and limitations of CBCT is essential for its appropriate application in the assessment and management of mandibular incisors.

Previous studies have demonstrated considerable variability in the prevalence and morphology of root canal configurations across different populations, suggesting that ethnic, geographic, and demographic factors may influence internal dental anatomy (Martins et al., 2017; Candeiro et al., 2019; Martins, Worldwide Anatomy Research Group & Versiani, 2023). Although the lingual canal has been investigated in several populations, evidence from Brazilian subpopulations remains limited, particularly regarding the combined assessment of prevalence, spatial characteristics, and demographic associations. Therefore, investigating these anatomical variations in a specific population may contribute to expanding current knowledge and improving the understanding of population-related differences in mandibular incisor anatomy.

Despite these advances, there remains a lack of large-sample studies addressing the prevalence and spatial characteristics of the lingual canal in mandibular incisors. It was hypothesized that these anatomical features vary according to demographic factors, particularly sex and age, and that CBCT imaging can reliably detect and characterize such variations under routine clinical conditions. Therefore, the aim of this study was to evaluate the prevalence, distribution, and general spatial characteristics of the lingual canal in mandibular incisors using CBCT images, as well as to investigate their association with sex and age. This study is clinically relevant, as it contributes to a better understanding of mandibular incisor anatomy and may help improve endodontic practice by reducing the risk of missed canals and treatment-related complications, under routine clinical conditions.

## Materials & Methods

### Study design and ethical authorization

This cross-sectional, retrospective, and quantitative observational study was conducted in accordance with the STROBE guidelines and followed the recommendations proposed in the Preferred Reporting Items for Epidemiologic Cross-sectional Studies on Root and Root Canal Anatomy Using Cone-beam Computed Tomographic Technology (Martins et al., 2020) to improve methodological transparency and reporting quality. Ethical approval was obtained from the Human Research Ethics Committee of Hermínio Ometto Foundation - FHO under approval number CAAE: 72550123.9.0000.5385. All procedures were conducted in accordance with the ethical principles of the Declaration of Helsinki and with Resolution No. 466/2012 of the Brazilian National Health Council governing research involving human participants.

### Sample size calculation

The sample was selected from an existing institutional CBCT database using a non-probabilistic consecutive sampling strategy. All available CBCT examinations performed during the study period were screened and consecutively assessed for eligibility according to the predefined inclusion and exclusion criteria. No additional randomization procedures were applied, as the study followed a retrospective observational design.

A sample size calculation was performed using G*Power software (version 3.1.9.4, Universität Kiel, Germany), based on statistical procedures and recent studies (Alfouzan et al., 2019; Kewalramani, Murthy & Gupta, 2019; Candeiro et al., 2019). Considering that the primary unit of analysis in this study was the tooth (mandibular incisor), the calculation was conducted at the tooth level.

For association tests, a minimum sample of 1,363 mandibular incisors was estimated, assuming a small effect size (*w* = 0.1), a significance level of 5%, a statistical power of 80%, and up to six degrees of freedom.

For multivariate analyses (MANOVA), the minimum required sample comprised 98 mandibular incisors presenting a second canal (lingual canal), based on a small effect size (f^2^(V) = 0.0625), a significance level of 5%, a statistical power of 80%, and a model including up to four groups, two predictors (sex and age), and two outcome variables (distance and angulation of spatial location).

Although analyses were conducted at the tooth level, the potential clustering of teeth within patients was considered during interpretation, given that multiple teeth from the same individual may share anatomical characteristics.

### Inclusion criteria

•  Patients with CBCT scans obtained for different clinical indications, including surgical planning, implant assessment, and endodontic evaluation;

•  CBCT scans presenting all four mandibular incisors within the field of view;

•  Patients aged 18 years or older, regardless of sex; and

•  Mandibular incisors with complete root development and sufficient image quality for anatomical assessment.

### Exclusion criteria

•  CBCT images with poor visualization quality or motion artifacts that impaired anatomical evaluation;

•  Patients undergoing orthodontic treatment or presenting implants affecting the mandibular incisor region;

•  Patients younger than 18 years;

•  Mandibular incisors with previous root canal treatment;

•  Teeth presenting root resorption, canal calcifications/obliteration, incomplete root development, or conditions compromising canal identification; and

•  Teeth with extensive restorative procedures or structural alterations that could interfere with the assessment of internal root canal anatomy.

The final study sample comprised 342 CBCT scans, including 211 female and 131 male patients. It was not possible to randomize patients according to sex, and all evaluated patients had four mandibular incisors, resulting in a total of 1,368 analyzed teeth.

### Procedures and measurements

The sociodemographic data and CBCT scans used in this study were obtained from the Radiology Center database of the School of Dentistry at CECAPE, located in Juazeiro do Norte, Ceará, Brazil. Institutional approval from CECAPE was obtained prior to the commencement of the research. Demographic variables included biological sex (female and male) and age. Age was analyzed both as a continuous variable and as a categorical variable, stratified into the following groups: <19, 20–29, 30–39, 40–49, 50–59, 60–69, and ≥70 years. The distribution of participants according to age group and sex is presented in Table 1.

**Table 1:** Demographic characteristics and occurrence of lingual canal in lower incisors in CBCT scans of the evaluated patients.

Variable	MD (SD) or n_i_ (%)	
	Female (n_i_= 211 (61.70))	Male (n_i_= 131 (38.30))
Average age (years)	51.12 ± 14.19	52.40 ± 14.47
Age range		
18 years old 20–29 years old 30–39 years old 40–49 years old 50–59 years old 60–69 years old ≥70 years	4 (1.90) 15 (7.10) 27 (12.80) 37 (17.55) 66 (31.30) 48 (22.75) 14 (6.60)	1 (0.76) 5 (3.82) 22 (16.79) 26 (19.85) 31 (23.66) 33 (25.19) 13 (9.92)
Total number of teeth evaluated		
Teeth 31 and 32 (left side) Teeth 41 and 42 (right side)	422 (50.00) 422 (50.00)	262 (50.00) 262 (50.00)
Occurrence of the lingual canal		
No Yes	145 (68.72) 66 (31.28)	76 (58.02) 55 (41.98)
Location of the lingual canal		
Teeth 31 and 32 (left side) Teeth 41 and 42 (right side)	32 (48.48) 34 (51.52)	20 (36.36) 35 (63.64)
Lingual canal position		
Straight Mesial Distal	49 (74.24) 8 (12,12) 9 (13.64)	40 (72.73) 6 (10.91) 9 (16.36)

**Notes.**

MD, Mean; SD, Standard deviation; n_i_, Absolute frequency; %, Percentage.

Following approval by the Research Ethics Committee, patients were contacted, informed about the study, and authorization was obtained to access their medical records stored at the institution. With guarantees of anonymity and confidentiality, all participants voluntarily agreed to participate. The Informed Consent Form (ICF) and Post-Informed Consent Form (PCF) were presented and signed by the participants, with additional clarification provided when necessary.

Image analyses were conducted between August 20 and October 20, 2023, by a single previously trained endodontist who was blinded to patient identity to minimize potential bias. All measurements were performed by the same examiner to ensure consistency and analytical reliability.

The CBCT examinations were performed using a Morita X800 F 150 unit (J. Morita Corp., Kyoto, Japan), operating at 100 kVp and 80 mA, with an exposure time of 17.86 s. All scans were acquired using standardized imaging parameters, including a limited field of view (FOV) of 10 × five cm, voxel size (slice thickness) of 0.5 mm, and a reconstruction interval of 1/1. The images were exported in DICOM format and subsequently analyzed using Dicom Viewer RadiAnt software (Medixant, Poznań, Poland). These acquisition parameters were maintained consistently across all examinations to ensure standardization of image quality and comparability of anatomical measurements.

To identify the location of the lingual canal, the first point at which the entrance of the second canal appeared in cross-sectional images of mandibular incisors was evaluated, progressing from the crown toward the root. Anatomical measurements were obtained using One Volume Viewer software (version 2.6.0, J. Morita Corporation) and included:

•  X distance: Distance between the buccal and lingual canals, expressed in millimeters;

•  Y angle: The midsagittal tomographic plane was used to identify incisor rotation in axial sections, using the center of the buccal canal as a reference point. This reference enabled precise identification of the incisor axis (line Y). The Y angle was defined as the angle between line X (connecting the buccal and lingual canals) and the incisor axis (line Y), with the vertex located at the center of the buccal canal.

**Figure 1:**
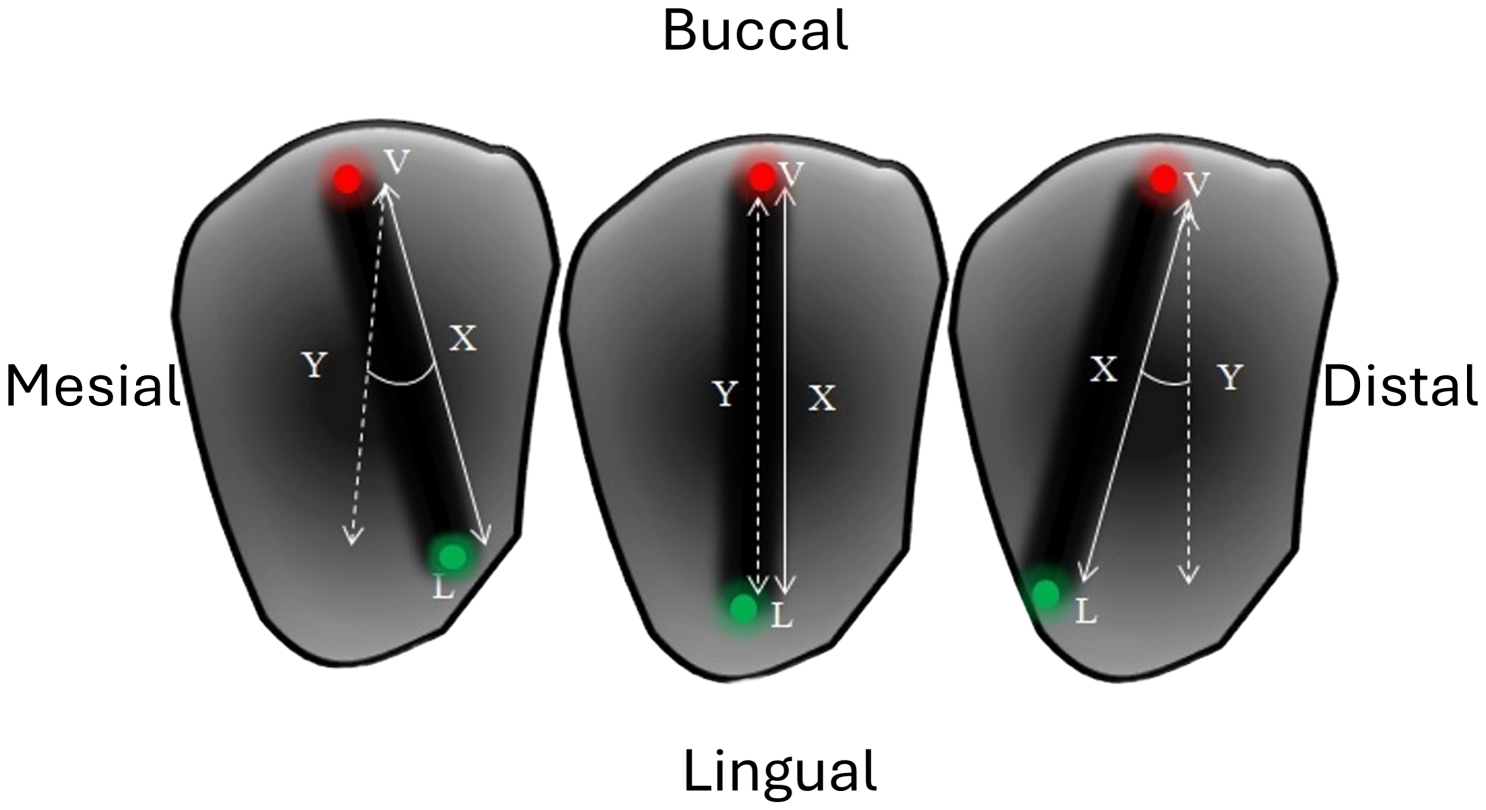
Schematic drawing of axial sections showing the linear distance between the buccal and lingual canals (X). Calculation of the angles between the buccal and lingual canals (Y).

Straight, mesial, and distal positions of the lingual canal were considered when measuring distances and angles (Fig. 1). All image analyses and measurements were performed by a single previously trained endodontist who was blinded to patient identity and demographic information to minimize assessment bias. Before formal evaluation, the examiner underwent calibration using the image analysis protocol and measurement procedures adopted in the study.

To assess reproducibility, intra-examiner agreement was evaluated using a test–retest design. A random subset corresponding to 30% of the total sample was reanalyzed by the same examiner after a 30-day interval under identical evaluation conditions. Agreement between repeated measurements was assessed using the Intraclass Correlation Coefficient (ICC), which demonstrated excellent reproducibility (ICC = 0.95), according to established interpretation criteria (Koo & Li, 2016). Since only one examiner performed all evaluations, interexaminer agreement analysis was not applicable.

### Statistical procedures

The analytical approach included descriptive statistics (means and standard deviations) for continuous variables and frequency distributions (absolute and relative) for categorical variables.

Prior to inferential analyses, assumptions of normality were assessed using the Shapiro–Wilk and Kolmogorov–Smirnov tests. Homogeneity of variances was evaluated using Levene’s test, and homogeneity of covariance matrices was assessed using Box’s M test (Box’s *M* = 20.95; F(15, 4188.42) = 1.26, *p* = 0.22). These assumptions supported the use of parametric procedures.

Associations between sociodemographic variables (sex and age group) and the presence of the lingual canal were assessed using Pearson’s chi-square test (*χ*^2^). Effect size was estimated using phi (*φ*) and Cramér’s V (*φ*c), interpreted according to conventional thresholds (small ≈ 0.10, moderate ≈ 0.30, large ≥ 0.50). For statistically significant associations, odds ratios (OR) and their respective 95% confidence intervals (95% CI) were calculated.

Differences in the spatial location of the lingual canal (distance and angle), according to sex and canal position, were evaluated using multivariate analysis of variance (MANOVA). Pillai’s Trace was adopted as the primary multivariate statistic due to its robustness. When appropriate, follow-up univariate analyses of variance (ANOVA) were performed. Effect sizes were expressed as partial eta squared (*η*^2^p) and interpreted as small (≈0.01), moderate (≈0.06), and large (≥0.14).

All analyses were performed using SPSS version 25 for Windows (IBM Corp., Armonk, NY, USA), with the level of significance set at *p* < 0.05.

## Results

The study included 342 CBCT scans (211 females and 131 males), totaling 1,368 mandibular incisors. Prevalence estimates were calculated at the tooth level (*n* = 1,368 mandibular incisors), whereas analyses investigating associations with demographic variables (sex and age group) were performed at the patient level (*n* = 342 CBCT examinations). Bilateral occurrence was evaluated descriptively according to the distribution of affected teeth and was not considered an independent analytical unit.

The mean age was 51.12 ± 14.19 years for females and 52.40 ± 14.47 years for males. The most frequent age group was 50–59 years for females (31.30%) and 60–69 years for males (25.19%) (Table 1).

A total of 121 teeth presented a lingual canal, corresponding to an overall prevalence of 8.84%. When stratified by sex, prevalence was higher in males (10.49%) than in females (7.82%).

Regarding laterality, the prevalence was 7.60% on the left side (teeth 31 and 32) and 10.08% on the right side (teeth 41 and 42). Among teeth presenting a lingual canal (*n* = 121), 57.02% were located in the right mandibular incisors (teeth 41 and 42), indicating a greater tooth-level occurrence on the right side. The straight configuration was the most common canal pattern, observed in both sexes.

Chi-square analysis revealed significant associations between lingual canal presence and sex (*χ*^2^(1) = 4.05, *p* < 0.05) and age group (*χ*^2^(6) = 17.24, *p* < 0.01) (Tables 2 and 3). Males were 1.59 times more likely to present a lingual canal than females. Adjusted residual analysis indicated that the age groups 40–49 and 50–59 years were significantly associated with the presence of a lingual canal.

**Table 2:** Association between the variables sex and occurrence of the lingual canal in incisor teeth in CBCT scans of the evaluated patients.

Variable	Occurrence of the lingual canal		*χ*^2^(*gl)*
	No (n_i_= 221 (64.62%))	Yes (n_i_= 121 (35.38%))	
Sex			
Female (n_i_(%)) Male (n_i_(%))	145 (68.72) 76 (58.02)	66 (31.28) 55 (41.98)	4.05 (1)^*^

**Notes.**

n_i_, Absolute frequency; %, Percentage; *χ*^2^, chi-square; df, degrees of freedom; ns, Not significant.

* *p* < 0.05.

**Table 3:** Association between the variables age group and the occurrence of the lingual canal in incisor teeth in CBCS scans of the evaluated patients.

Occurrence of the lingual canal	Age range (years)							*χ*^2^(gl)
	<19	20–29	30–39	40–49	50–59	60–69	≥70	
No (n _i_ (%))	3 (1.36)	13 (5.88)	26 (11.76)	30 (13.57)	72 (32.58)	59 (26.70)	18 (8.14)	17.24 (6)^**^
Adjusted residuals	−0.20 ^ns^	0.00 ^ns^	−1.80 ^ns^	−3.10^**^	2.30^**^	1.80 ^ns^	0.20 ^ns^	
Yes (n_i_ (%))	2 (1.65)	7 (5.79)	23 (19.01)	33 (27.27)	25 (20.66)	22 (18,18)	9 (7.44)	
Adjusted residuals	0.20 ^ns^	0.00 ^ns^	1.80 ^ns^	3.10^**^	2.30^**^	1.80 ^ns^	0.20 ^ns^	

**Notes.**

n_i_, Absolute frequency; %, Percentage; *χ*^2^, chi-square; df, degrees of freedom; ns, Not significant.

** *p* < 0.01.

Multivariate analysis of variance (MANOVA) was performed to evaluate differences in the spatial location of the lingual canal (distance and angle) according to sex and canal position. The results showed no statistically significant multivariate effect of sex (Pillai’s Trace = 0.004, F(2,114) = 0.247, *p* = 0.782, *η*^2^p = 0.004), canal position (Pillai’s Trace = 0.011, F(4,230) = 0.330, *p* = 0.858, *η*^2^p = 0.006), or their interaction (Pillai’s Trace = 0.018, F(4,230) = 0.531, *p* = 0.713, *η*^2^p = 0.009).

Follow-up univariate ANOVA analyses confirmed that there were no significant differences in canal spatial location according to sex or canal position (distance: *F* = 1.06, *p* = 0.35; angle: *F* = 0.01, *p* = 0.99).

**Figure 2:**
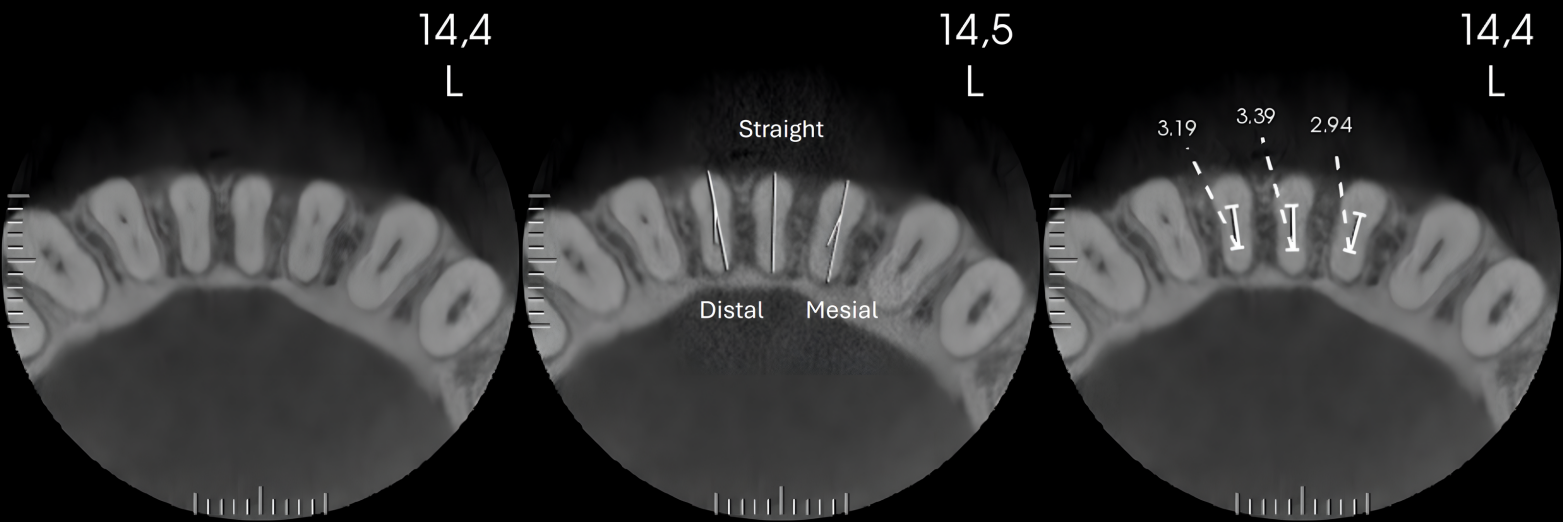
Illustrative CBCT images of randomly selected patient to simulate the evaluated angle (straight, mesial and distal) and distance (in mm) between the buccal and lingual canals.

Descriptive analysis demonstrated minimal variation in canal distance and angle between groups. Mean distances ranged from 2.27 to 2.44 mm in females and from 2.25 to 2.62 mm in males, while angles ranged from 27.17° to 27.51° in females and from 27.88° to 28.07° in males (Table 4, Fig. 2).

**Table 4:** Descriptive statistics and results for the variables - spatial location (distance and angle) of the lingual canal, biological sex, and position of the lingual canal in incisor teeth in CBCT scans of the evaluated patients.

Variable	Sex	Lingual canal position	MD (DP)	F (*gl)*	*p*-value
Distance	Female	Straight	2.33 (0.66)		
		Mesial	2.44 (0.49)		
		Distal	2.27 (0.69)		
		Total	2.33 (0.64)	1.06 (2)^ns^	0.35
	Male	Straight	2.25 (0.52)		
		Mesial	2.36 (0.14)		
		Distal	2.62 (0.45)		
		Total	2.32 (0.49)		
Angle	Female	Straight	27.25 (4.45)		
		Mesial	27.51 (6.14)		
		Distal	27.17 (6.79)		
		Total	27.27 (4.94)	0.01 (2)^ns^	0.99
	Male	Straight	28.07 (4.19)		
		Mesial	27.99 (4.69)		
		Distal	27.88 (5.02)		
		Total	28.03 (4.29)		

**Notes.**

MD, Mean; SD, Standard deviation; *F*, F test; ns, not significant.

## Discussion

The evaluation of the prevalence of the lingual canal in mandibular incisors is clinically relevant, as it may directly influence endodontic procedures. This study assessed the occurrence of this anatomical variation in 1,368 mandibular incisors using CBCT and analyzed its association with patient sex and age in a sample of 342 individuals from Juazeiro do Norte, Ceará, Brazil.

CBCT is a well-established imaging modality that enables detailed three-dimensional assessment of root canal anatomy. It allows accurate identification of root canal configurations, supports access cavity planning, and improves the detection of additional canals and anatomical complexities, thereby enhancing the effectiveness of endodontic treatment (La et al., 2010; Zhang et al., 2011; Ferrari, Abu Hasna & Martinho, 2021).

The overall prevalence of the lingual canal observed in this study (8.84%) differs from that reported in previous CBCT-based investigations. For instance, Mashyakhy (2019) reported substantially higher values, with a prevalence of 26.3% in mandibular central incisors and 30.8% in lateral incisors (28.6% overall). These discrepancies likely reflect both demographic and methodological sources of variability. Population-related factors, including genetic background, ethnicity, age distribution, and sex composition, may influence root canal morphology and contribute to differences in the prevalence of lingual canals among populations (Martins et al., 2017; Candeiro et al., 2019; Kewalramani, Murthy & Gupta, 2019). At the same time, methodological aspects such as CBCT acquisition protocols, voxel size, image reconstruction parameters, eligibility criteria, and analytical unit selection (tooth-level *versus* patient-level analysis) may substantially affect the sensitivity for detecting anatomical variations and consequently influence prevalence estimates. Multicenter investigations have demonstrated wide variability in reported prevalence rates—from less than 10% to over 30%, highlighting that comparisons across studies should consider both population characteristics and methodological heterogeneity (Martins, Worldwide Anatomy Research Group & Versiani, 2023).

Patient-related factors, such as sex and age, may also be associated with root canal morphology. In the present study, male patients showed a higher prevalence of the lingual canal compared to females, consistent with findings reported in different populations (Mashyakhy, 2019; Martins et al., 2017; Lin et al., 2014; Martins, Worldwide Anatomy Research Group & Versiani, 2023).

Age-related analysis revealed a higher probability of lingual canal presence in individuals aged 40–59 years. In contrast, other studies have reported peak prevalence in younger age groups (21–40 years) (Martins, Worldwide Anatomy Research Group & Versiani, 2023). These differences may be associated with age-related changes in dentin deposition, such as secondary dentin formation (Kewalramani, Murthy & Gupta, 2019). However, given the cross-sectional design of the present study, these findings should be interpreted as associations within the studied population rather than evidence of causal or developmental changes. Clinically, this highlights the importance of considering patient age during treatment planning.

At the tooth level, a greater occurrence of lingual canals was observed in right mandibular incisors. However, this finding should be interpreted descriptively, as bilateral occurrence was not modeled as an independent outcome and no inference regarding patient-level laterality can be established. Previous studies have reported either a left-side predominance (Saati et al., 2018) or no significant difference between sides (Martins, Worldwide Anatomy Research Group & Versiani, 2023). These findings emphasize the variability of mandibular incisor anatomy and the importance of individualized assessment.

Recognition of anatomical symmetry and asymmetry is clinically important, as it may affect treatment outcomes. A careful and individualized approach is essential to minimize the risk of missed canals and procedural errors (Mashyakhy, 2019).

Regarding canal configuration, the straight lingual canal was the most prevalent pattern, regardless of sex. This may facilitate detection and instrumentation, reducing the risk of procedural errors such as ledging or canal transportation. In contrast, mesial or distal deviations may increase technical difficulty and the risk of iatrogenic complications (Prado et al., 2023; Srivastava, 2024). No significant differences in canal position were observed according to sex or canal configuration.

The findings of this study reinforce the clinical importance of identifying lingual canal variations in mandibular incisors. Consideration of patient-specific factors, such as sex and age, may support a more individualized approach, improve treatment accuracy, and reduce the risk of complications. From a diagnostic perspective, CBCT remains a valuable tool for detecting these anatomical variations (Martins, Worldwide Anatomy Research Group & Versiani, 2023).

This study has some limitations. Its retrospective cross-sectional design may introduce selection bias, as it relies on pre-existing CBCT scans that may not fully represent the general population. Although all evaluations were performed by a single trained examiner to ensure consistency, the inclusion of multiple examiners would require detailed calibration procedures to ensure reproducibility.

Importantly, the voxel size used (0.5 mm) represents a relatively low spatial resolution for assessing fine endodontic anatomical structures. This may have reduced the sensitivity for detecting small or narrow lingual canals, particularly those with minimal separation from the main canal, potentially leading to an underestimation of their true prevalence. Previous studies have demonstrated that smaller voxel sizes improve the detection of additional canals and subtle anatomical variations (Kamburoğlu et al., 2014; Vizzotto et al., 2013).

However, it is important to note that CBCT scans with similar voxel sizes have been widely used in anatomical studies of mandibular incisors (Candeiro et al., 2019; Mashyakhy, 2019), supporting the clinical applicability of this imaging protocol. Furthermore, larger voxel sizes are commonly adopted in clinical settings to balance image quality and radiation dose. Therefore, despite this limitation, the present findings remain clinically relevant and reflective of real-world diagnostic conditions.

This methodological consideration is further supported by recent evidence comparing CBCT with micro-computed tomography (micro-CT), which remains the reference standard for detailed assessment of root canal anatomy. Recent studies have demonstrated that although CBCT provides clinically valuable three-dimensional information, its diagnostic performance for detecting accessory canals and fine anatomical structures is lower than that of micro-CT and strongly influenced by imaging resolution parameters (voxel size and acquisition settings). Therefore, prevalence estimates derived from CBCT should be interpreted as clinically applicable anatomical observations rather than absolute anatomical prevalence values. Nonetheless, CBCT remains the most feasible noninvasive method for *in vivo* assessment and epidemiological investigation of root canal morphology in clinical populations (Pires et al., 2024).

Finally, the sample was restricted to a Brazilian sub-population from the Northeast region, which may limit the generalizability of the findings. Future studies should include more diverse populations and explore additional factors influencing root canal morphology. A deeper understanding of these variations may contribute to improved endodontic outcomes and more personalized patient care.

## Conclusions

The present findings indicate that, in this sample, the lingual canal was more frequently observed in mandibular incisors of male patients, in individuals aged 40–59 years, and on the right side. The straight configuration was the most observed canal pattern. These results contribute to the characterization of anatomical variation in mandibular incisors and may support clinical awareness during endodontic assessment; however, they should be interpreted within the observational design of the study and the limitations of the imaging protocol. Further studies using higher-resolution imaging and more diverse populations are recommended to improve the understanding of these anatomical variations and their potential clinical relevance.

## Supplemental Information

10.7717/peerj.21617/supp-1 Supplemental Information 1 Demographic characteristics and the distance and angulation of each evaluated dental root

10.7717/peerj.21617/supp-2 Supplemental Information 2 Presence or absence of lingual canal in each evaluated patient

10.7717/peerj.21617/supp-3 Supplemental Information 3 STROBE checklist

## Additional Information and Declarations

### Data Availability

The following information was supplied regarding data availability: The raw measurements are available in the Supplemental Files.

## References

[ref-1] Abu Hasna A, Pereira Santos D, Gavlik De Oliveira TR, Pinto ABA, Pucci CR, Lage-Marques JL (2020). Apicoectomy of perforated root canal using bioceramic cement and photodynamic therapy. International Journal of Dentistry.

[ref-2] Ahmed HMA, Wolf TG, Rossi-Fedele G, Dummer PMH (2024). The study and relevance of pulp chamber anatomy in endodontics - a comprehensive review. European Endodontic Journal.

[ref-3] Alfouzan K, Alfadley A, Alkadi L, Alhezam A, Jamleh A (2019). Detecting the second mesiobuccal canal in maxillary molars in a saudi arabian population: a micro-CT study. Scanning.

[ref-4] Al-Nahlawi T, Ala Rachi M, Abu Hasna A (2021). Endodontic perforation closure by five mineral oxides silicate-based cement with/without collagen sponge matrix. International Journal of Dentistry.

[ref-5] Assadian H, Dabbaghi A, Gooran M, Eftekhar B, Sharifi S, Shams N, Dehghani Najvani A, Tabesh H (2016). Accuracy of CBCT, digital radiography and cross-sectioning for the evaluation of mandibular incisor root canals. Iranian Endodontic Journal.

[ref-6] Baxter S, Jablonski M, Hülsmann M (2020). Cone-beam-computed-tomography of the symmetry of root canal anatomy in mandibular incisors. Journal of Oral Science.

[ref-7] Candeiro GT De M, Gonçalves SDS, Lopes LL De A, Lima IT De F, Alencar PNB, Iglecias EF, Silva PGB (2019). Internal configuration of maxillary molars in a subpopulation of Brazil’s Northeast region: a CBCT analysis. Brazilian Oral Research.

[ref-8] Castillo Marin MC, Abu Hasna A, Frozoni M, Diamantino MGG, Rocha CT, Valera MC, Carvalho CAT (2024). Prodesign logic files effect on apical foramen wear and shape transformation after foraminal enlargement. Dentistry Journal.

[ref-9] Costa FFNP, Pacheco-Yanes J, Siqueira JF, Oliveira ACS, Gazzaneo I, Amorim CA, Santos PHB, Alves FRF (2019). Association between missed canals and apical periodontitis. International Endodontic Journal.

[ref-10] Dos Reis FAS, Abu Hasna A, Ragozzini G, De Moura FB, Campos TMB, De Martin AS, Carvalho CAT, Bueno CES (2023). Assessing the cyclic fatigue resistance and sterilization effects on replica-like endodontic instruments compared to Reciproc Blue. Scientific Reports.

[ref-11] Ferrari CH, Hasna AAbu, Martinho FC (2021). Three Dimensional mapping of the root apex: distances between apexes and anatomical structures and external cortical plates. Brazilian Oral Research.

[ref-12] Henrique Ferrari C, Abu Hasna A (2022). Panoramic radiography and cone-beam computed tomography to measure distances between root apexes and anatomical structures. Journal of Oral Research.

[ref-13] Kamburoğlu K, Murat S, Kılıç C, Yüksel S, Avsever H, Farman A, Scarfe WC (2014). Accuracy of CBCT images in the assessment of buccal marginal alveolar peri-implant defects: effect of field of view. Dentomaxillofacial Radiology.

[ref-14] Kewalramani R, Murthy CS, Gupta R (2019). The second mesiobuccal canal in three-rooted maxillary first molar of Karnataka Indian sub-populations: a cone-beam computed tomography study. Journal of Oral Biology and Craniofacial Research.

[ref-15] Koo TK, Li MY (2016). A guideline of selecting and reporting intraclass correlation coefficients for reliability research. Journal of Chiropractic Medicine.

[ref-16] Krasner P, Rankow HJ (2004). Anatomy of the pulp-chamber floor. Journal of Endodontics.

[ref-17] La S-H, Jung D-H, Kim E-C, Min K-S (2010). Identification of independent middle mesial canal in mandibular first molar using cone-beam computed tomography imaging. Journal of Endodontics.

[ref-18] Lee J-B, Seo M-S (2022). Mandibular incisors with two canals are associated with the presence of the distolingual root in mandibular first molars: a cone-beam computed tomographic study. BMC Oral Health.

[ref-19] Lin Z, Hu Q, Wang T, Ge J, Liu S, Zhu M, Wen S (2014). Use of CBCT to investigate the root canal morphology of mandibular incisors. Surgical and Radiologic Anatomy.

[ref-20] Martins JNR, Kishen A, Marques D, Nogueira Leal Silva EJ, Caramês J, Mata A, Versiani MA (2020). Preferred reporting items for epidemiologic cross-sectional studies on root and root canal anatomy using cone-beam computed tomographic technology: a systematized assessment. Journal of Endodontics.

[ref-21] Martins JNR, Marques D, Mata A, Caramês J (2017). Root and root canal morphology of the permanent dentition in a Caucasian population: a cone-beam computed tomography study. International Endodontic Journal.

[ref-22] Martins JNR, Versiani MA, Worldwide Anatomy Research Group (2023). Worldwide prevalence of the lingual canal in mandibular incisors: a multicenter cross-sectional study with meta-analysis. Journal of Endodontics.

[ref-23] Mashyakhy M (2019). Anatomical analysis of permanent mandibular incisors in a Saudi Arabian population: an in Vivo cone-beam computed tomography study. Nigerian Journal of Clinical Practice.

[ref-24] Paes da Silva Ramos Fernandes LM, Rice D, Ordinola-Zapata R, Capelozza ALAlvares, Bramante CM, Jaramillo D, Christensen H (2014). Detection of various anatomic patterns of root canals in mandibular incisors using digital periapical radiography, 3 cone-beam computed tomographic scanners, and micro-computed tomographic imaging. Journal of Endodontics.

[ref-25] Pires M, Martins JNR, Pereira MR, Vasconcelos I, da CostaRP, Duarte I, Ginjeira A (2024). Diagnostic value of cone beam computed tomography for root canal morphology assessment - a micro-CT based comparison. Clinical Oral Investigations.

[ref-26] Prado HS, Petean IBF, Franco NJS, Camargo RV, Carvalho KKT, Mazzi-Chaves JF, Lopes-Olhê FC, Silva-Sousa YTC, Souza-Gabriel AE, Sousa-Neto MD (2023). Impact of access cavities on root canal preparation, restorative protocol quality, and fracture resistance of teeth. Brazilian Oral Research.

[ref-27] Ragozzini G, Abu Hasna A, Dos Reis FAS, De Moura FB, Campos TMB, Bueno CES, Carvalho CAT, De Martin AS (2024). Effect of autoclave sterilization on the number of uses and resistance to cyclic fatigue of WaveOne gold and four replica-like endodontic instruments. International Journal of Dentistry.

[ref-28] Rahimi S, Milani AS, Shahi S, Sergiz Y, Nezafati S, Lotfi M (2013). Prevalence of two root canals in human mandibular anterior teeth in an Iranian population. Indian Journal of Dental Research.

[ref-29] Saati S, Shokri A, Foroozandeh M, Poorolajal J, Mosleh N (2018). Root morphology and number of canals in mandibular central and lateral incisors using cone beam computed tomography. Brazilian Dental Journal.

[ref-30] Srivastava S (2024). Root canal instrumentation: current trends and future perspectives. Cureus.

[ref-31] Vizzotto MB, Silveira PF, Arús NA, Montagner F, Gomes BPFA, Da Silveira HED (2013). CBCT for the assessment of second mesiobuccal (MB2) canals in maxillary molar teeth: effect of voxel size and presence of root filling. International Endodontic Journal.

[ref-32] Zhang R, Yang H, Yu X, Wang H, Hu T, Dummer PMH (2011). Use of CBCT to identify the morphology of maxillary permanent molar teeth in a Chinese subpopulation. International Endodontic Journal.

