# Supplementary material for: Prevalence and morphology of lingual canals in mandibular incisors: a cone-beam computed tomography-based cross-sectional study
Source: PeerJ. 2026 Aug 5;14:e21617. doi: 10.7717/peerj.21617 (PMC13452421; doi:10.7717/peerj.21617)
Supplement: Supplementary file 1 [file peerj-14-21617-s003.pdf]

STROBE Statement—checklist of items that should be included in reports of observational studies

	Item No.	Recommendation	Page No.	Relevant text from manuscript
**Title and abstract**	1	(*a*) Indicate the study's design with a commonly used term in the title or the abstract	4	Line 1-3
		(*b*) Provide in the abstract an informative and balanced summary of what was done and what was found	4	Line. 31-35 and Line 49-52
**Introduction**				
Background/rationale	2	Explain the scientific background and rationale for the investigation being reported	6	Line 99-103
Objectives	3	State specific objectives, including any prespecified hypotheses	6	Line 96-99
**Methods**				
Study design	4	Present key elements of study design early in the paper	6	Line 108-109
Setting	5	Describe the setting, locations, and relevant dates, including periods of recruitment, exposure, follow-up, and data collection	8	Line 142-145 and Line 152
Participants	6	(*a*) *Cohort study*—Give the eligibility criteria, and the sources and methods of selection of participants. Describe methods of follow-up	-	-
		Case-control study—Give the eligibility criteria, and the sources and methods of case ascertainment and control selection. Give the rationale for the choice of cases and controls	-	-
		Cross-sectional study—Give the eligibility criteria, and the sources and methods of selection of participants	7	Line 123-140
		(*b*) *Cohort study*—For matched studies, give matching criteria and number of exposed and unexposed	-	-
		Case-control study—For matched studies, give matching criteria and the number of controls per case	-	-
Variables	7	Clearly define all outcomes, exposures, predictors, potential confounders, and effect modifiers. Give diagnostic criteria, if applicable	8	Line 156-165
Data sources/ measurement	8*	For each variable of interest, give sources of data and details of methods of assessment (measurement). Describe comparability of assessment methods if there is more than one group	8	Line 166-173
Bias	9	Describe any efforts to address potential sources of bias	8 and 9	Line 152-155 and Line 175-182
Study size	10	Explain how the study size was arrived at	7	Line 117--121

Continued on next page

Quantitative variables	11	Explain how quantitative variables were handled in the analyses. If applicable, describe which groupings were chosen and why	9	Line 185-187
Statistical methods	12	(*a*) Describe all statistical methods, including those used to control for confounding	9	Line 188-198
		(*b*) Describe any methods used to examine subgroups and interactions	9	Line 188-198
		(*c*) Explain how missing data were addressed	N/A	N/A
		(*d*) *Cohort study*—If applicable, explain how loss to follow-up was addressed<br>*Case-control study*—If applicable, explain how matching of cases and controls was addressed<br>*Cross-sectional study*—If applicable, describe analytical methods taking account of sampling strategy	-<br>-<br>9	-<br>-<br>Line 188-200
		(*e*) Describe any sensitivity analyses	9	Line 175-182
**Results**				
Participants	13*	(a) Report numbers of individuals at each stage of study—eg numbers potentially eligible, examined for eligibility, confirmed eligible, included in the study, completing follow-up, and analysed	9	Line 204-205
		(b) Give reasons for non-participation at each stage	N/A	N/A
		(c) Consider use of a flow diagram	N/A	N/A
Descriptive data	14*	(a) Give characteristics of study participants (eg demographic, clinical, social) and information on exposures and potential confounders	9 and 10	
		(b) Indicate number of participants with missing data for each variable of interest	N/A	N/A
		(c) *Cohort study*—Summarise follow-up time (eg, average and total amount)	-	-
Outcome data	15*	*Cohort study*—Report numbers of outcome events or summary measures over time	-	-
		Case-control study—Report numbers in each exposure category, or summary measures of exposure	-	-
		Cross-sectional study—Report numbers of outcome events or summary measures	10 and 11	Line 217-222 and Line 251-261
Main results	16	(*a*) Give unadjusted estimates and, if applicable, confounder-adjusted estimates and their precision (eg, 95% confidence interval). Make clear which confounders were adjusted for and why they were included	10 and 11	Line 223-250
		(*b*) Report category boundaries when continuous variables were categorized	N/A	N/A
		(*c*) If relevant, consider translating estimates of relative risk into absolute risk for a meaningful time period	10 and 11	Line 223-250

Continued on next page

Other analyses	17	Report other analyses done—eg analyses of subgroups and interactions, and sensitivity analyses	10 and 11	Line 223-250
**Discussion**				
Key results	18	Summarise key results with reference to study objectives	12	Line 276-289
Limitations	19	Discuss limitations of the study, taking into account sources of potential bias or imprecision. Discuss both direction and magnitude of any potential bias	14	Line 345-354
Interpretation	20	Give a cautious overall interpretation of results considering objectives, limitations, multiplicity of analyses, results from similar studies, and other relevant evidence	13 and 14	Line 323-345
Generalisability	21	Discuss the generalisability (external validity) of the study results	14	Line 355-362
**Other information**				
Funding	22	Give the source of funding and the role of the funders for the present study and, if applicable, for the original study on which the present article is based	N/A	N/A

*Give information separately for cases and controls in case-control studies and, if applicable, for exposed and unexposed groups in cohort and cross-sectional studies.

Note: An Explanation and Elaboration article discusses each checklist item and gives methodological background and published examples of transparent reporting. The STROBE checklist is best used in conjunction with this article (freely available on the Web sites of PLoS Medicine at http://www.plosmedicine.org/, Annals of Internal Medicine at http://www.annals.org/, and Epidemiology at http://www.epidem.com/). Information on the STROBE Initiative is available at www.strobe-statement.org.
